# Control of translation elongation in health and disease

**DOI:** 10.1242/dmm.043208

**Published:** 2020-03-26

**Authors:** John R. P. Knight, Gavin Garland, Tuija Pöyry, Emma Mead, Nikola Vlahov, Aristeidis Sfakianos, Stefano Grosso, Fabio De-Lima-Hedayioglu, Giovanna R. Mallucci, Tobias von der Haar, C. Mark Smales, Owen J. Sansom, Anne E. Willis

**Affiliations:** 1 Beatson Institute for Cancer Research, Glasgow G61 1BD, UK; 2 MRC Toxicology Unit, University of Cambridge, Lancaster Road, Leicester LE1 9HN, UK; 3 School of Biosciences, University of Kent, Canterbury, Kent CT2 7NJ, UK; 4 UK Dementia Research Institute at the University of Cambridge and Department of Clinical Neurosciences, University of Cambridge, Cambridge CB2 0XY, UK; 5 Institute of Cancer Sciences, University of Glasgow, Glasgow, G61 1QH, UK

**Keywords:** Elongation control, Protein synthesis, mRNA translation control

## Abstract

Regulation of protein synthesis makes a major contribution to post-transcriptional control pathways. During disease, or under stress, cells initiate processes to reprogramme protein synthesis and thus orchestrate the appropriate cellular response. Recent data show that the elongation stage of protein synthesis is a key regulatory node for translational control in health and disease. There is a complex set of factors that individually affect the overall rate of elongation and, for the most part, these influence either transfer RNA (tRNA)- and eukaryotic elongation factor 1A (eEF1A)-dependent codon decoding, and/or elongation factor 2 (eEF2)-dependent ribosome translocation along the mRNA. Decoding speeds depend on the relative abundance of each tRNA, the cognate:near-cognate tRNA ratios and the degree of tRNA modification, whereas eEF2-dependent ribosome translocation is negatively regulated by phosphorylation on threonine-56 by eEF2 kinase. Additional factors that contribute to the control of the elongation rate include epigenetic modification of the mRNA, coding sequence variation and the expression of eIF5A, which stimulates peptide bond formation between proline residues. Importantly, dysregulation of elongation control is central to disease mechanisms in both tumorigenesis and neurodegeneration, making the individual key steps in this process attractive therapeutic targets. Here, we discuss the relative contribution of individual components of the translational apparatus (e.g. tRNAs, elongation factors and their modifiers) to the overall control of translation elongation and how their dysregulation contributes towards disease processes.

## Abstract (precis)

**Summary:** In this Review, we outline the process of elongation and discuss the relative contributions of transfer RNAs, elongation factors and their modifiers to this process, and how their dysregulation contributes towards disease.

## Introduction

**Fig. 1.**
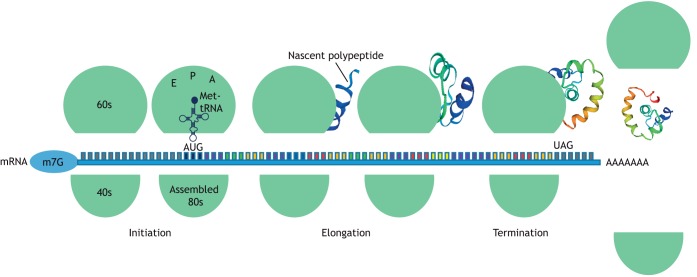
**A schematic to represent the three-stage process of mRNA translation.** Translation initiation involves the assembly of elongation-competent ribosomes [shown in green and containing three tRNA-binding sites called the aminoacyl (A), peptidyl (P) and exit (E) sites] in which an initiator Met-tRNA base-pairs with the initiation codon in the ribosomal P site. In general, initiation of translation is controlled by the bioavailability of eukaryotic initiation factors, RNA-binding proteins that aid the recruitment of the mRNA to the ribosome and RNA regulatory motifs. Elongation rates are regulated by the availability of tRNAs, the codon sequence of the message, the modifications to the coding sequence and the activity of eEF2, with some messages additionally requiring eIF5A. Met-tRNA, methionine transfer RNA; m7G, 7-methylguanosine; 40s, small ribosomal subunit; 60s, large ribosomal subunit; 80s, ribosome.

Protein synthesis can be considered a three-stage process of initiation, elongation and termination (Fig. 1). Data from a number of teams have shown that the elongation stage is highly regulated, which is perhaps unsurprising given that this step of mRNA translation consumes almost all the energy utilised during protein synthesis (Proud, 2015). However, compared to regulation of the initiation of translation, elongation control has been much less studied. Understanding the regulation of translation elongation is essential, however, since it is now well established that dysregulation of this process contributes to disease, e.g. cancers and neurodegeneration (Faller et al., 2015; Jan et al., 2018; Beckelman et al., 2019).

**Fig. 2.**
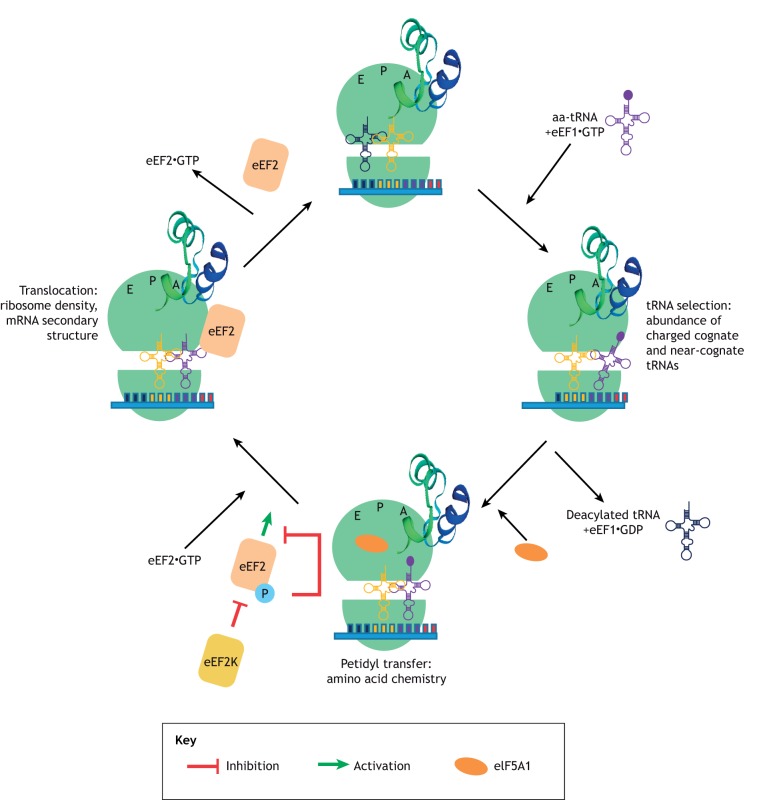
**A schematic to represent the process of elongation.** The four basic steps are shown. The ribosome contains three tRNA-binding sites: the aminoacyl (A), peptidyl (P) and exit (E) sites. In the first step of peptide elongation, the tRNA, which is in a complex with eIF1 and GTP and contains the cognate anticodon to the mRNA coding sequence, enters the A site. Recognition of the tRNA leads to the hydrolysis of GTP and eviction of eEF1 from the A site. In parallel, the deacylated tRNA in the E site is ejected. The A site and the P site tRNAs interact, which allows ribosome-catalysed peptide bond formation to take place. This involves the transfer of the polypeptide to the aa-tRNA, thus extending the nascent polypeptide by one amino acid. eIF5A allosterically assists in the formation of certain peptide bonds, e.g. proline-proline. eEF2 then enters the A site and, through the hydrolysis of GTP, induces a change in the ribosome conformation and stimulates translocation. The ribosome is then in a correct conformation to accept the next aa-tRNA and commence another cycle of elongation.

In the elongation phase, the mRNA is decoded by the ribosome in a process that requires selection of each aminoacyl-transfer RNA (aa-tRNA), which is dictated by the mRNA codon in the ribosome acceptor (A) site, peptide bond formation and movement of both tRNAs and the mRNA through the ribosome (Voorhees and Ramakrishnan, 2013) (Fig. 2). A new amino acid is incorporated into a nascent peptide at a rate of approximately one every sixth of a second (Voorhees and Ramakrishnan, 2013; Choi et al., 2018). The first step of this process requires guanosine triphosphate (GTP)-bound eukaryotic elongation factor 1A (eEF1A) to recruit an aa-tRNA to the aminoacyl (A) site, which has an anticodon loop cognate to the codon sequence of the mRNA (Choi et al., 2018). The anticodon of this sampling tRNA does not initially base-pair with the A-site codon. Instead, the tRNA dynamically remodels to generate a codon-anticodon helix (Voorhees and Ramakrishnan, 2013), which stabilises the binding of the tRNA-eEF1A-GTP complex to the ribosome A site (Rodnina et al., 2005). This helical structure is energetically favourable for cognate or correct pairing, and so discriminates between the non-cognate or unpaired and single mismatched or near-cognate species (Choi et al., 2018). This is important for the accuracy of decoding since it provides a mechanism to reject a non-cognate tRNA that carries an inappropriate amino acid (Plant et al., 2007). The pairing of the tRNA and codon induces GTP hydrolysis by eEF1A, which is then evicted from the A site. In parallel with this process, the ribosome undergoes a conformational change that stimulates contact between the 3′ end of the aa-tRNA in the A site and the tRNA carrying the polypeptide chain in the peptidyl (P) site. The shift in position of the two tRNAs [A to the P site and P to the exit (E) site] results in ribosome-catalysed peptide bond formation and the transfer of the polypeptide to the aa-tRNA, thus extending the polypeptide by one amino acid (Moazed and Noller, 1989). The second stage of the elongation cycle requires a GTPase, eukaryotic elongation factor 2 (eEF2), which enters the A-site and, through the hydrolysis of GTP, induces a change in the ribosome conformation. This stimulates ribosome translocation to allow the next aa-tRNA to enter the A-site (Voorhees and Ramakrishnan, 2013; Choi et al., 2018), thus starting a new cycle of elongation (Fig. 2). In this Review, we discuss how the process of elongation is controlled, the relative contributions of the individual components to this process, how they are in turn regulated and how their dysregulation is associated with disease, particularly cancers and neurodegeneration.

## Control of tRNA-dependent decoding and its role in disease

A number of factors influence the rate at which elongation proceeds. As discussed in detail below, decoding speeds are affected by the relative abundance of each tRNA, the cognate:near-cognate tRNA ratios (Sabi and Tuller, 2014; Fluitt et al., 2007; Tarrant and Von Der Haar, 2014), the extent of tRNA modification [particularly at the uridine-34 (U34) wobble position] and the degree of tRNA aminoacylation (Rezgui et al., 2013; Ranjan and Rodnina, 2017). Elongation rates also depend on the activity of eEF2, which, as discussed above, is required for ribosome translocation, and which is negatively regulated by phosphorylation on threonine-56 (T56) (Ryazanov and Davydova, 1989; Proud, 2015). Additional factors that also contribute to the control of the elongation rate include epigenetic modification of the mRNA coding sequence (Hanson et al., 2018; Presnyak et al., 2015; Radhakrishnan et al., 2016) and the expression levels of proteins with a role in elongation. This includes eukaryotic translation initiation factor 5A (eIF5A), which is required to stimulate peptide bond formation between proline residues, whose rigid structures would otherwise impact negatively on the elongation process (reviewed in Dever et al., 2014).

### tRNA abundance

tRNA abundance is crucial for cell homeostasis. Functional tRNA pool availability can be controlled at the level of transcription, maturation, modification and charging. The human genome contains ∼500 tRNA genes, of which ∼300 are expressed (Chan and Lowe, 2016; Schimmel, 2018). As the genetic code is degenerate, each amino acid can be encoded by two, four or six synonymous three-nucleotide codons (with the exception of methionine and tryptophan). Synonymous codons encoding the same amino acid are used unevenly within a transcriptome, in a mechanism conserved across organisms and termed ‘codon usage bias’ (Spencer et al., 2012). Importantly, tRNA genes show differential tissue expression (Dittmar et al., 2006; Gingold et al., 2014), which mirrors codon usage bias. In this case, synonymous codons are over- or under-represented in relation to the mRNAs that encode tissue-specific proteins, which in turn control the expression levels of such proteins. Thus, large relative differences in protein expression are achieved when using luciferase variants that have been codon optimised, according to the tRNA abundance in specific mammalian cell lines (Xie et al., 2018). Moreover, in addition to tissue-specific differences in tRNA expression levels, two distinct tRNA expression profiles have been observed that correspond to pro-proliferation or pro-differentiation codon usage signatures (Gingold and Pilpel, 2011; Gingold et al., 2014). Taken together, these data indicate that tRNA expression is tightly linked to cell-type-specific mRNA expression. This is particularly the case in tumour-derived cells. While cancer cells require elevated levels of tRNAs to respond to increased protein synthesis rates (Pavon-Eternod et al., 2009; Zhou et al., 2009), the data demonstrate that they also selectively reprogramme the tRNA expression profile to favour codon usage required by a subset of cancer-related genes (Gingold et al., 2014). This enhances their translation and, in turn, promotes metastasis (Pavon-Eternod et al., 2009; Goodarzi et al., 2015). For example, genetically engineered overexpression of the initiator methionine tRNA in the mouse promotes angiogenesis in tumour-associated fibroblasts and metastasis of melanoma tumour cells (Clarke et al., 2016; Birch et al., 2016). In contrast, mouse models have also shown that increased bulk tRNA synthesis driven by overexpression of the RNA polymerase III subunit Brf1 had little effect in a model of pancreatic tumorigenesis (Liko et al., 2019), suggesting that specific tRNA reprogramming may play a greater role than total tRNA expression levels in driving this cancer. However, Brf1 overexpression in a mouse model of prostate cancer accelerates the disease, indicating that increases in tRNA expression may promote certain tumours, although it is possible that this phenotype is driven by upregulation of specific tRNAs (Loveridge et al., 2019). Importantly, the tRNAs induced in cancer cells favour a pro-proliferative programme and their upregulation is associated with a worse prognosis (Gingold et al., 2014; Hernandez-Alias et al., 2019).

### tRNA wobble position and decoding

Certain codons can be decoded not only by cognate, but also by near-cognate tRNAs. This flexibility is achieved via a mismatch between the third base of the codon and the first base of the anticodon, the ‘wobble’ position (Plant et al., 2007). In the human genome, 14 tRNA anti-codons are decoded via wobble base-pairing (Chan and Lowe, 2016), and some of these wobble-decoded codons are decoded more slowly than the synonymous Watson-Crick-decoded codons, due to a higher probability of incorrect rejection of wobble-decoding tRNAs by the ribosome (Kothe and Rodnina, 2007). In agreement with this, ribosome occupancy times obtained from foot-printing data using human cell lines shows increased ribosome occupancy at wobble codons (Richter and Coller, 2015; Stadler and Fire, 2011).

**Fig. 3.**
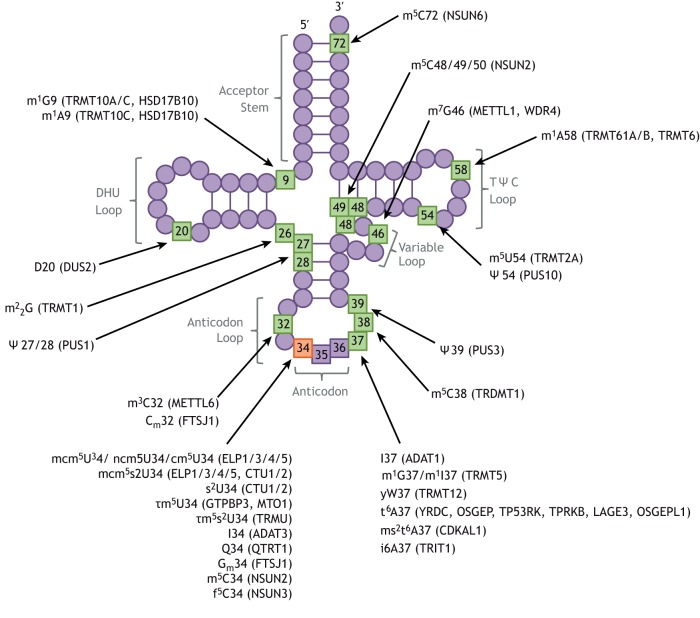
**Schematic representation of human tRNA.** Residues with post-transcriptional modifications associated with human diseases are shown in green and the wobble base (34) is shown in orange. The annotations explain the disease-associated tRNA modification with the known protein(s) required for the tRNA modifications shown in brackets (de Crécy-Lagard et al., 2019): A, adenosine; C, cytidine; C_m_, 2′-O-methylcytidine; cm^5^U, 5-carboxymethyl uridine; D, dihydrouridine; f^5^C, 5-formylcytidine; G, guanosine; G_m_, 2′-O-methylguanosine; I, inosine; i6A, N6-isopentenyladenosine; m^1^, 1-methyl; m^2^_2_G, N(2),N(2)-dimethylguanosine; m^3^, 3-methyl; m^5^, 5-methyl; m^7^, 7-methyl; mcm^5^s^2^U, 5-methoxycarbonylmethyl-2-thiouridine; mcm^5^U, 5-methoxycarbonylmethyluridine; ms^2^t^6^A, 2-methylthio-N(6)-threonylcarbamoyladenosine; ncm^5^U, 5-carbamoylmethyluridine; Q, queosine; s^2^U, 2-thiouridine; t^6^A, N(6)-threonylcarbamoyladenosine; U, uridine; yW, wybutosine; Ψ, pseudouridine; τm^5^U, 5-taurinomethyluridine; τm^5^s^2^U, 5-taurinomethyl-2-thiouridine.

There are ∼100 different post-transcriptional modifications of RNAs and the majority of these occur on tRNAs (Fig. 3). On average, there are 13 modifications per tRNA molecule, although this varies, e.g. tRNA^His^ is less modified, whereas tRNA^Tyr^ is possibly the most modified tRNA (El Yacoubi et al., 2012; de Crécy-Lagard et al., 2019). Such modifications are an essential part of the maturation process to generate functional tRNA molecules (El Yacoubi et al., 2012; de Crécy-Lagard et al., 2019). They are crucial to the stability, folding and decoding properties of tRNAs and in some cases regulate tRNA charging, e.g. binding to aminoacyl-tRNA synthetases (ARSs), the enzymes responsible for charging amino acids to their cognate tRNAs (Schimmel, 2018; de Crécy-Lagard et al., 2019). Modifications in the anticodon loop mainly affect decoding and translational efficiency. Of particular importance is U34 of the tRNA anticodon, which base-pairs with the wobble position of the codon and displays a wide variety of chemical modifications essential for near-cognate binding (Phizicky and Hopper, 2010; Grosjean and Westhof, 2016). This position is frequently modified in tRNAs that decode split codon-box amino acids, in which the A- and G-ending codons encode a different amino acid than the U- and C-ending codons (Phizicky and Hopper, 2010; Grosjean and Westhof, 2016). An *in vitro* study showed that U34 modifications in tRNA^Lys(UUU)^ and in tRNA^Gln(UUG)^ increased the affinity of the tRNA to its cognate codon in the A site of the ribosome (Rezgui et al., 2013; Ranjan and Rodnina, 2017) and therefore allowed faster translation. In addition, many human tRNAs that have an A at the wobble base have a modification from A to I (inosine), which allows base pairing from A34-U to I34-U, I34-C or, in some cases, even to I34-A, increasing the encoding capacity of the modified tRNAs (Novoa et al., 2012; Pan, 2018).

Enzymes that catalyse U34 modifications have been implicated in cancer and neurological diseases (Close et al., 2018). For example, the Elongator complex is required for a subset of tRNA modifications at U34 to promote efficient decoding of G- and A-ending codons. It comprises six functionally essential subunits, which include Elongator complex protein 1 (ELP1; also known as IKAP and IKBKAP) and the enzymatic component Elongator complex protein 3 (ELP3) (Huang et al., 2005; Johansson et al., 2008). Mutations in Elongator complex components have been associated with various neurological diseases, including familial dysautonomia (Karlsborn et al., 2014; Dalwadi and Yip, 2018). In this condition, mutations in *ELP1* reduce neuronal expression of full-length ELP1 protein and decrease U34 modification (Karlsborn et al., 2014; Anderson et al., 2001; Slaugenhaupt et al., 2001; Dong et al., 2002; Cuajungco et al., 2003; Yoshida et al., 2015). Consequently, this results in a decoding deficiency, codon-specific ribosome pausing and frameshifting, leading to the accumulation of misfolded proteins (Rezgui et al., 2013; Tükenmez et al., 2015; Klassen et al., 2017; Nedialkova and Leidel, 2015). In mice, this activates the PKR-like endoplasmic reticulum (ER) kinase (PERK) pathway within the unfolded protein response, a cellular response to restore ER homeostasis in response to protein misfolding (recently reviewed in Pizzinga et al., 2019), which interferes with the differentiation of cortical progenitors and neurogenesis in the developing cortex by promoting the generation of intermediate progenitors (Laguesse et al., 2015; Rezgui et al., 2013). The changes in decoding efficiency might also drive an alternative translational programme that favours specific codon subsets that perpetuate the disease state (Rezgui et al., 2013; Fernández-Vázquez et al., 2013; Kapur and Ackerman, 2018). Thus, Elongator-dependent tRNA modification plays critical roles in defining the balance between proliferation and differentiation in developing neural tissues, disruption of which is likely to play a causal role in the pathogenesis of various neurological diseases (Laguesse et al., 2015).

Elongator-dependent tRNA modifications are also dysregulated in cancers with elevated ELP3 expression, such as WNT-dependent colon cancer, breast cancer and *BRAF^V600E^* melanoma. In these cancers, ELP3-mediated tRNA modification directs a codon-specific translational programme. This promotes efficient translation of transcripts that encode critical effectors of the oncogenic state, such as SRY-box transcription factor 9 (Sox9) in colon cancer, DEK in breast cancer and hypoxia-inducible factor 1-alpha (HIF1α) in *BRAF^V600E^* melanoma, to drive tumour initiation, survival, metastasis and drug resistance (Ladang et al., 2015; Delaunay et al., 2016; Rapino et al., 2018). In contrast, low ELP3 expression is reportedly a factor of poor prognosis for endometrioid adenocarcinoma (Wang et al., 2012). Hence, the role of Elongator-dependent tRNA modifications in cancer appear to be context dependent, but represent a promising opportunity for therapeutic intervention in some neoplastic diseases.

### tRNA aminoacylation

In order for a tRNA to be functional, it must first be charged with its corresponding amino acid at its 3′ end by the appropriate ARS. Reports suggest that >80% of tRNA isodecoders, which share a common anticodon, but differ elsewhere in their sequence, in human cells are charged, with the exception of tRNA^Ser^ and tRNA^Thr^ isodecoders, which are charged between 60% and 80% (Evans et al., 2017). Aminoacylation controls the distribution of charged tRNAs in the cell and therefore defects in this process can cause highly abundant tRNAs to become functionally limited. Consequently, mutations in genes that encode ARSs have been associated with an array of human diseases, including neurological disorders, with recessive and dominant phenotypes (Meyer-Schuman and Antonellis, 2017). Indeed, neurons are particularly vulnerable to translation elongation defects and the consequent proteostatic disruption due to the specialised translational burden required to facilitate synaptic plasticity and high metabolic activity and restricted ability to dilute misfolded proteins through cell division (Kapur et al., 2017; March et al., 2019). While mutations in mitochondrial ARS genes predominantly affect tissues with high metabolic activity such as the central nervous system, autosomal recessive mutations in cytoplasmic ARS genes typically affect a wider range of tissues, which usually include a neurological component (Meyer-Schuman and Antonellis, 2017). In contrast, a smaller number of ARS genes have been implicated in dominant neuropathic disorders with a limited phenotypic range, most notably Charcot-Marie-Tooth (CMT) disease (Meyer-Schuman and Antonellis, 2017).

Recessive ARS mutant-associated disease pathogenesis is likely caused by reductions in ARS protein levels and activity, resulting in deficient charging of specific tRNAs, which could directly cause ribosome stalling or frame shifting (Meyer-Schuman and Antonellis, 2017; Zhang et al., 2010). Intriguingly, some ARS mutations result in tissue-specific phenotypes, which might either indicate that mutations in a specific ARS could modulate the translation of cell-type-specific transcripts in a codon-dependent manner or expose tissue-specific deficiencies in active tRNA pools (Meyer-Schuman and Antonellis, 2017; Puffenberger et al., 2012). Abnormal expression levels of different ARS genes have also been reported in several cancers, but their pathogenic role has thus far only been linked to non-canonical activities, such as signalling in oncogenic pathways (Kushner et al., 1976; Scandurro et al., 2001; Wasenius et al., 2003; Park et al., 2005; Tzima and Schimmel, 2006; Vellaichamy et al., 2009; Kim et al., 2011; Kim et al., 2012).

In contrast, the pathogenesis of dominant ARS mutant disease is possibly dictated by a dominant-negative mechanism whereby levels of charged tRNA are depleted below a haploinsufficient state through oligomerisation of a subfunctional ARS protein with wild-type subunits (Malissovas et al., 2016). An alternative dominant toxic gain-of-function effect for mutant glycine(G)ARS has been proposed, perturbing neuronal signalling via interactions with neuropilin-1 (NRP1) and neurotrophic receptor tyrosine kinase 1/2/3 (NTRK1/2/3; also known as TrkA/B/C) (He et al., 2015; Sleigh et al., 2017). However, it is not clear whether either of these potential mechanisms apply to all neuropathy-associated ARS mutations, and research into a unifying mechanism of ARS-mediated disease pathology remains ongoing (Meyer-Schuman and Antonellis, 2017; Yao and Fox, 2013).

### Control of elongation by eEF2K and eEF2, and disease status

Phosphorylation of eEF2 on threonine-56 inhibits its activity by physically blocking entry into the A site, thereby reducing ribosome translocation and the elongation rate (Proud, 2015). The enzyme required for this phosphorylation is eEF2 kinase (eEF2K; a calcium/calmodulin-dependent member of the α-kinase group). eEF2K, which appears to be non-essential under physiological conditions, is activated following nutrient and energy depletion to slow down the rate of elongation (Proud, 2015). Interestingly, while eEF2K knockout protected mice from a lethal dose of whole-body ionizing radiation at 8 Gy by reducing apoptosis in the gastrointestinal tract, it was not protective at 20 Gy, which causes severe damage to the gastrointestinal tract and increases mitotic cell death in small intestinal stem cells (Liao et al., 2016). Taken together, these data suggest that eEF2K provides a protective strategy in time of cells stress, but that this effect is cell-type dependent. This is an important point when considering whether eEF2K may be a useful target in the treatment of disease.

### eEF2K in tumorigenesis

The role of eEF2K in cancer development has been studied extensively; however, there is no current consensus as to whether eEF2K is beneficial or detrimental to cancer cell survival. This has led to the hypothesis that eEF2K could act as a ‘double-edged sword’, with cancer-cell-type-specific functions (Liu and Proud, 2016). This is exemplified by data that correlate either high or low eEF2K expression with improved patient prognosis (Table 1).

**Table 1.**
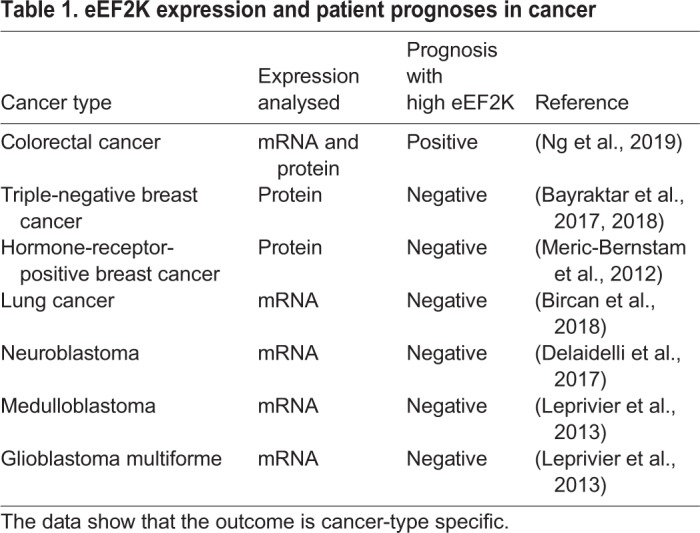
**eEF2K expression and patient prognoses in cancer**

The promotion of cancer cell survival by eEF2K has been linked to nutrient management in rapidly proliferating solid tumours, where, due to the poor vascular structure, cancer cells have to adapt to prolonged nutrient deprivation. Thus, inhibition of elongation via eEF2K, and therefore energy conservation, enhanced the survival of oncogene-transformed fibroblasts (Leprivier et al., 2013). Moreover, similar data were obtained in an orthotopic model of breast cancer using RNA interference (RNAi) suppression of eEF2K (Tekedereli et al., 2012), and by pharmacological inhibition of eEF2K in triple-negative murine breast cancer cells (Liu et al., 2014). Importantly, these studies are supported by clinical data showing a negative correlation between eEF2K expression and patient prognosis in medulloblastoma, glioblastoma multiforme (Leprivier et al., 2013) and breast cancer (Table 1).

eEF2K is also required to maintain energy production during acidosis and hypoxia, when it significantly reduces ATP levels and induces cancer cell death (Xie et al., 2015). Similarly, eEF2K knockdown was shown to generate acute sensitivity to oxidative stress following suppression of the tumour suppressor p53 homologue TAp73 (Marini et al., 2018). Loss of TAp73 leads to a remarkable eEF2K-dependent translation elongation blockade, resulting in sensitivity to oxidative stress due to impaired translation of mitochondrial proteins (Marini et al., 2018). Thus, eEF2K acts downstream of TAp73 to allow translational reprogramming and adaptation to oxidative stress, thereby enhancing cancer cell survival. Additionally, eEF2K may actively participate in rerouting metabolic pathways in cancer. eEF2K maintains the expression of the pyruvate kinase isoform PK-M2 (also known as PKM), which promotes the switch to Warburg metabolism in cancer cells (Cheng et al., 2016). However, it remains to be determined how important the regulation of PK-M2 is to the eEF2K-dependent response to nutrient deprivation or stress.

eEF2K is also important for cancer cell migration and metastasis. A recent study that focused on the migratory and metastatic potential of cancer cells showed that knockdown or chemical inhibition of eEF2K led to reduced integrin expression and decreased migration and invasion both *in vitro* and *in vivo* (Xie et al., 2018). These data are supported by previous studies in which inhibition or genetic ablation of eEF2K suppressed cancer cell migration and invasion (Bayraktar et al., 2017; Zhu et al., 2017).

Suppression of eEF2K can also promote tumorigenesis, with a dramatic increase in cell proliferation driven by the elevated elongation speed. In a mouse model of intestinal cancer driven by loss of the adenomatous polyposis coli (*Apc*) gene, activation of mechanistic target of rapamycin complex 1 (mTORC1) drives increased translational elongation; however, this can be successfully targeted with rapamycin to inhibit intestinal tumorigenesis (Faller et al., 2015). The pharmacological targeting of mTORC1 required functional eEF2K, suggesting that mTORC1 promotes intestinal cancer by repressing eEF2K. Thus, low eEF2K activity is required for rapid proliferation, which is consistent with low eEF2K correlating with poor survival in colorectal cancer patients (Ng et al., 2019).

In addition, there are data to suggest that the benefits of low eEF2K activity in cancer may not be limited to colorectal cancer. In tumour-derived cell lines, including HeLa, MCF7 and A549, as well as Ras^V12^-transformed mouse embryonic fibroblast (MEF) lines, the anti-viral drug nelfinavir was shown to activate eEF2K independently of mTORC1 inhibition or 5′ adenosine monophosphate-activated protein kinase (AMPK), leading to the phosphorylation of eEF2, decreased protein synthesis and increased cancer cell death (De Gassart et al., 2016). eEF2K was required for all nelfinavir-mediated anti-tumour activity, indicating suppression of elongation to be the drug's mechanism of action.

Collectively, studies of eEF2K in cancer have revealed some vulnerabilities that may allow targeting of this pathway for therapeutic benefit. Conflicting mechanistic data as to whether eEF2K promotes or suppresses tumour growth expand this therapeutic potential further, as the pathway could be susceptible to either activation or suppression. The key goal now is to discover which tumour types, or indeed subtypes, are susceptible to what mode of eEF2K targeting.

### Dysregulation of eEF2 function and neurodegenerative disease

The role of eEF2 phosphorylation in the homeostatic brain is linked to the Ca^2+^ sensitivity of eEF2K, which controls local protein synthesis rates upon ion influx from neurotransmission (reviewed in Taha et al., 2013; Delaidelli et al., 2019). Here, we focus on the role of this pathway in neurodegeneration. Both eEF2 and eEF2K are expressed within the axons, dendrites and soma of neurons and show aberrant expression in neurodegenerative pathologies. *eEF2K* mRNA expression is increased in Alzheimer's disease (AD) brains, and eEF2 phosphorylated T56 (T56-P) is heightened in the hippocampus and cortex of AD patients, in close proximity to areas of deposition of Tau. This protein is the main component of the intracellular filamentous inclusions that result in AD-associated proteotoxicity (Jan et al., 2017; Beckelman et al., 2019). Similarly, in Parkinson's disease (PD), *eEF2K* shows higher levels of transcription, and eEF2 T56-P is increased in neurons within, but not limited to, regions affected by the disease (Jan et al., 2018). Importantly, mouse models of both AD and PD show increased phosphorylation of eEF2 (Ma et al., 2014; Jan et al., 2018; Beckelman et al., 2019). Genetic or pharmacological targeting of eEF2K in AD or PD cultured neurons, *Caenorhabditis* *elegans* or mouse models (Ma et al., 2014) restores normal electrophysiology and even reverses disease-associated behavioural defects (Ma et al., 2014; Jan et al., 2018; Jan et al., 2017). Interestingly, eEF2K inhibition does not correlate with altered deposition of amyloid plaques (Beckelman et al., 2019), but instead reduces reactive oxygen species within cultured neurons and increases synapse formation (Jan et al., 2018; Beckelman et al., 2019).

Modulation of eEF2K activity, in addition to its effects on global protein synthesis rates, also selectively controls the expression of subsets of neuroprotective proteins, although this is context dependent. For example, heterozygous deletion of eEF2K in the Tg19959 AD mouse model showed increased expression calbindin in the hippocampi, which corresponded with neuroprotection (Beckelman et al., 2019). However, in healthy neurons, phosphorylation of eEF2 and a decrease in elongation rates rapidly increased translation of synaptic proteins with potentially neuroprotective functions such as brain-derived neurotrophic factor (BDNF) and tripartite motif containing 3 (TRIM3) in response to normal synaptic activity (Autry et al., 2011; Heise et al., 2016; Verpelli et al., 2010). Moreover, in mice with early-stage prion disease, cooling-associated suppression of elongation via phosphorylation of eEF2K correlated with increased expression of the synaptic protein reticulon 3 (RTN3), which was shown to be neuroprotective (Bastide et al., 2017). RTN3 overexpression was mediated by the interaction of the corresponding *Rtn3* transcript with RNA-binding protein 3 (RBM3), which promoted its recruitment to the ribosome, but was also driven by the decrease in elongation rate (Bastide et al., 2017; Peretti et al., 2015).

But how does suppression of global translation elongation result in enhanced expression of select transcripts? These can be explained in a model in which the relative rates of the two steps of translation elongation, decoding and translocation, govern specific gene expression on a global scale (Bastide et al., 2017). Transcripts that allow fast decoding due to their usage of highly abundant tRNAs are more responsive to changes in the rate of translocation than transcripts that are decoded more slowly. Thus, the slowest-decoded messages escape suppression by slowing of translocation, whereas the faster-decoded messages are repressed. eEF2 catalyses translocation, with its phosphorylation slowing this step of elongation to make decoding the limiting step across the majority of messages. *RTN3* is decoded by lower-abundance tRNAs, meaning that its expression is increased due to insensitivity to suppressed translocation. It would be of interest to assess the role of RNA-binding proteins, such as RBM3, and our decoding/translocation rate model on the expression of additional transcripts that are induced by eEF2K activation in neurons, such as calbindin, BDNF and TRIM3 (Autry et al., 2011; Heise et al., 2016; Verpelli et al., 2010).

eEF2 is of great interest in neuron biology and pathology, with studies of its roles in neurodegenerative diseases helping to reveal previously unappreciated mechanisms of gene expression control. Extensive preclinical work presents eEF2K as an attractive therapeutic target for both AD and PD, especially given the viability of eEF2K knockout and kinase-impaired mice (Gildish et al., 2012; Chu et al., 2014b), although these mice display reduced female fertility at advanced age and impaired learning. However, the importance of eEF2 phosphorylation in the normal brain has also been documented (Autry et al., 2011; Heise et al., 2016), illustrating the need for the cautious application of eEF2K inhibitors against neurodegenerative diseases.

## Additional factors that contribute to elongation rates

### eIF5A

Although eIF5A was originally described as a translation initiation factor (Benne and Hershey, 1978; Schreier et al., 1977; Kemper et al., 1976), its role in translation elongation and termination account for most of its activity (Schuller et al., 2017; Pelechano and Alepuz, 2017). Humans have two isoforms of eIF5A – eIF5A1 and eIF5A2 – that share 84% sequence similarity (Gordon et al., 1987), with eIF5A1 being widely expressed and eIF5A2 only present in the brain and testis (Jenkins et al., 2001; Clement et al., 2003). eIF5A is the only protein to contain hypusine. Hypusination of eIF5A is a two-step process through which deoxyhypusine synthase catalyses the addition of an aminobutyl moiety from spermidine to the ε-amino group of lysine 50 of eIF5A, resulting in the formation of deoxyhypusine-eIF5A. Subsequently, deoxyhypusine hydroxylase hydroxylates deoxyhypusine-eIF5A to form the mature hypusine-eIF5A (Cooper et al., 1983; Park et al., 2010). This post-translational modification of eIF5A is essential for its function since lysine mutants lack activity (Cano et al., 2008).

eIF5A binds in the E-site of the ribosome and projects the hypusine-containing domain towards the P-site where it binds the CCA-end of the tRNA (Fig. 2) and allosterically assists in the formation of certain peptide bonds, e.g. proline-proline, to prevent ribosomal stalling on these motifs (Gutierrez et al., 2013; Schuller et al., 2017; Saini et al., 2009). For example, in B cells, eIF5A is required for the efficient elongation on poly-proline stretches of transcription factor EB (TFEB) (Zhang et al., 2019). Several recent studies have identified multiple other tripeptides that require eIF5A function, including DDP, PDP and DNP and non-proline containing tri-peptides such as RDK, DVG and DDG, revealing a broader role of eIF5A in translation elongation (Schuller et al., 2017). In addition to its function in elongation, a recent study has also shown that eIF5A is important for start codon selection since depletion of this protein leads to an increase in translation via upstream open-reading frames (Manjuanth et al., 2019).

eIF5A has been associated with development and progression of multiple types of cancer (Mathews and Hershey, 2015). Recent data, which describe a role of this protein in the modulation of mitochondrial function, apoptosis and autophagy, are consistent with a pro-proliferative and pro-survival function (Puleston et al., 2019; Zhang et al., 2019; Lubas et al., 2018). Taken together, these data suggest that eIF5A could be a promising new target for the development of drugs targeting specific subtypes of cancer.

### Coding sequences

The mRNA sequence within coding regions influences both elongation rates and mRNA stability. Studies in yeast and other organisms have shown that mRNA stability is directly regulated by elongation speed, with slow decoding being sensed by the decay machinery, meaning that non-optimal codons confer higher instability on mRNA sequences than optimal codons (Hanson et al., 2018; Presnyak et al., 2015; Radhakrishnan et al., 2016). Similarly, recent data have shown that this codon-dependent translational effect on mRNA stability is conserved in human cells and depends on the number of ribosomes translating a given mRNA (Narula et al., 2019; Wu et al., 2019).

### mRNA modification

Advances in studying the epitranscriptome have identified modifications within mRNA coding regions that influence translation efficiency (Arango et al., 2018; Dominissini et al., 2012). Intriguingly, N4-acetylcytidine (ac4C) modification of mRNAs primarily occurs within coding sequences and is strongly enriched for cytidine within wobble positions. When compared to unmodified cytidine, ac4C enhances thermal stability of Watson-Crick base-pairing, therefore promoting correct decoding and enhancing both translation and mRNA stability (Arango et al., 2018). These observations raise the possibility that modifications within the mRNA coding sequence represent an emerging gene expression control mechanism at the level of translation.

### Crosstalk between elongation and initiation of mRNA translation

Although initiation and elongation are considered to be distinct processes, some lines of evidence suggest that inhibition of elongation, which leads to ribosome pausing or stalling, feeds back to inhibit initiation, thus linking the regulation of these two steps in protein synthesis. First, codon-dependent ribosome movement around the start codon affects translation initiation rates (Chu et al., 2014a). Second, blocking hypusination and, thereby, the activity of eIF5A results in a total loss of polysomes and a defect in translation initiation, rather than only an elongation block (Mandal et al., 2013). Third, the eIF2α kinase general control non-derepressible 2 (GCN2), which inhibits the formation of the ternary complex, comprised of eIF2, GTP and tRNA_i_^Met^, and required to bring the initiator tRNA to the ribosome, is associated with the ribosome complex known as the P-stalk. The P-stalk contains a GTPase-associated centre in which eEF1A and eEF2 function, suggesting that GCN2 may be able to sense stalled ribosomes (Inglis et al., 2019), although further experimentation is required to prove this hypothesis. Finally, new research in MEFs shows that hydrogen peroxide treatment induces phosphorylation of eEF2, which, in turn, results in the phosphorylation of eIF2α and inhibition of initiation (Sanchez et al., 2019). Taken together, these data suggest a mechanism by which acute inhibition of translation is initiated by blocking elongation, either through eEF2 phosphorylation or reduced expression and activity of eIF5A, whereas eIF2α phosphorylation is required to maintain the longer-term suppression of protein synthesis.

## Conclusions

Control of translation elongation is essential to maintain protein and cellular homeostasis, and there is strong evidence demonstrating that dysregulation of this process is associated with disease such as cancer and neurodegenerative disorders, as discussed herein, but also with cardiovascular disease (reviewed in Liu and Proud, 2016). There are a number of stages at which elongation rates can be manipulated to block disease progression. These include the inhibition of enzymes that are required for tRNA modifications at the wobble base U34, the selective targeting of specific ARSs, interfering with eIF5A function through inhibition of hypusination and modulating the activity of eEF2K. In particular, eEF2K is an attractive target since it is not required for mammalian development or maintenance of cell viability, suggesting that its inhibition may not have deleterious effects in healthy tissue. Moreover, this protein is very distinct from other mammalian kinases and thus it may be amenable to inhibition by selective compounds that do not target other protein kinases. However, while inhibition of eEF2K may be therapeutically beneficial in blocking cancer cell survival and restoring protein synthesis in AD, further studies are required to define its role in different cancer types and at differing stages in tumorigenesis to fully assess its utility as a therapeutic target in oncology. Overall, defining new ways to modify elongation control will lead to the development of new therapeutic targets, although more detailed studies are required to fully understand the role of this process in cell-type-specific regulation of protein synthesis.
